# Supplementary material for: Modeling glioblastoma heterogeneity as a dynamic network of cell states
Source: Mol Syst Biol. 2021 Sep 16;17(9):e10105. doi: 10.15252/msb.202010105 (PMC8444284; doi:10.15252/msb.202010105)
Supplement: Supplementary file 6 — Source Data for Figure 5 [file MSB-17-e10105-s004.zip › Figure5A_sourcedata/GSEA_3071/hallmarks_stateIII.GseaPreranked.1623742540963/HALLMARK_UV_RESPONSE_DN.html]

Details for gene set HALLMARK\_UV\_RESPONSE\_DN[GSEA]

|  || Dataset | state33071 |
| Phenotype | NoPhenotypeAvailable |
| Upregulated in class | na\_neg |
| GeneSet | HALLMARK\_UV\_RESPONSE\_DN |
| Enrichment Score (ES) | -0.43130553 |
| Normalized Enrichment Score (NES) | -2.2677636 |
| Nominal p-value | 0.0 |
| FDR q-value | 0.0013221734 |
| FWER p-Value | 0.008 |
Table: GSEA Results Summary

  

Fig 1: Enrichment plot: HALLMARK\_UV\_RESPONSE\_DN      
 Profile of the Running ES Score & Positions of GeneSet Members on the Rank Ordered List

  

| PROBE | GENE SYMBOL | GENE\_TITLE | RANK IN GENE LIST | RANK METRIC SCORE | RUNNING ES | CORE ENRICHMENT || 1 | MYC |  |  | 599 | -0.260 | -0.1439 | No |
| 2 | CDK13 |  |  | 779 | -0.268 | -0.1791 | No |
| 3 | BDNF |  |  | 806 | -0.270 | -0.1749 | No |
| 4 | VAV2 |  |  | 963 | -0.277 | -0.2039 | No |
| 5 | SCHIP1 |  |  | 995 | -0.279 | -0.2005 | No |
| 6 | PMP22 |  |  | 1023 | -0.281 | -0.1961 | No |
| 7 | LAMC1 |  |  | 1120 | -0.286 | -0.2093 | No |
| 8 | TFPI |  |  | 1141 | -0.287 | -0.2028 | No |
| 9 | ATP2B4 |  |  | 1347 | -0.297 | -0.2436 | No |
| 10 | PEX14 |  |  | 1415 | -0.301 | -0.2487 | No |
| 11 | CDON |  |  | 1423 | -0.301 | -0.2383 | No |
| 12 | INPP4B |  |  | 1618 | -0.313 | -0.2756 | No |
| 13 | WDR37 |  |  | 1919 | -0.331 | -0.3395 | No |
| 14 | MET |  |  | 2138 | -0.344 | -0.3817 | No |
| 15 | INSIG1 |  |  | 2266 | -0.354 | -0.4001 | No |
| 16 | DLG1 |  |  | 2317 | -0.359 | -0.3984 | No |
| 17 | MAP2K5 |  |  | 2358 | -0.362 | -0.3941 | No |
| 18 | GJA1 |  |  | 2362 | -0.362 | -0.3802 | No |
| 19 | HAS2 |  |  | 2400 | -0.365 | -0.3749 | No |
| 20 | SDC2 |  |  | 2537 | -0.376 | -0.3947 | No |
| 21 | ERBB2 |  |  | 2680 | -0.389 | -0.4155 | Yes |
| 22 | ID1 |  |  | 2685 | -0.389 | -0.4008 | Yes |
| 23 | PRKCE |  |  | 2692 | -0.390 | -0.3865 | Yes |
| 24 | PLCB4 |  |  | 2735 | -0.394 | -0.3814 | Yes |
| 25 | FHL2 |  |  | 2851 | -0.403 | -0.3947 | Yes |
| 26 | LDLR |  |  | 2880 | -0.406 | -0.3854 | Yes |
| 27 | ABCC1 |  |  | 2943 | -0.414 | -0.3847 | Yes |
| 28 | SLC22A18 |  |  | 2971 | -0.416 | -0.3748 | Yes |
| 29 | BHLHE40 |  |  | 2981 | -0.417 | -0.3602 | Yes |
| 30 | SMAD3 |  |  | 3091 | -0.430 | -0.3708 | Yes |
| 31 | NFIB |  |  | 3102 | -0.431 | -0.3559 | Yes |
| 32 | RASA2 |  |  | 3295 | -0.459 | -0.3868 | Yes |
| 33 | TENT4A |  |  | 3297 | -0.459 | -0.3685 | Yes |
| 34 | PLPP3 |  |  | 3439 | -0.490 | -0.3849 | Yes |
| 35 | SERPINE1 |  |  | 3474 | -0.499 | -0.3734 | Yes |
| 36 | TGFBR3 |  |  | 3546 | -0.525 | -0.3704 | Yes |
| 37 | NRP1 |  |  | 3562 | -0.532 | -0.3528 | Yes |
| 38 | ATP2B1 |  |  | 3580 | -0.539 | -0.3353 | Yes |
| 39 | AMPH |  |  | 3629 | -0.565 | -0.3248 | Yes |
| 40 | PDGFRB |  |  | 3678 | -0.598 | -0.3129 | Yes |
| 41 | COL5A2 |  |  | 3726 | -0.636 | -0.2992 | Yes |
| 42 | MMP16 |  |  | 3753 | -0.675 | -0.2786 | Yes |
| 43 | F3 |  |  | 3764 | -0.693 | -0.2531 | Yes |
| 44 | EFEMP1 |  |  | 3784 | -0.716 | -0.2289 | Yes |
| 45 | CELF2 |  |  | 3822 | -0.774 | -0.2071 | Yes |
| 46 | COL11A1 |  |  | 3828 | -0.788 | -0.1764 | Yes |
| 47 | IGFBP5 |  |  | 3895 | -1.082 | -0.1496 | Yes |
| 48 | COL1A1 |  |  | 3902 | -1.191 | -0.1028 | Yes |
| 49 | COL1A2 |  |  | 3906 | -1.278 | -0.0518 | Yes |
| 50 | COL3A1 |  |  | 3918 | -1.418 | 0.0028 | Yes |
Table: GSEA details [plain text format]

  

Fig 2: HALLMARK\_UV\_RESPONSE\_DN: Random ES distribution      
 Gene set null distribution of ES for **HALLMARK\_UV\_RESPONSE\_DN**
